# Estrogen receptor beta inhibits transcriptional activity of hypoxia inducible factor-1 through the downregulation of arylhydrocarbon receptor nuclear translocator

**DOI:** 10.1186/bcr2854

**Published:** 2011-03-24

**Authors:** Wonchung Lim, Yeomyung Park, Jungyoon Cho, Choa Park, Joonwoo Park, Young-Kwon Park, Hyunsung Park, YoungJoo Lee

**Affiliations:** 1 Department of Bioscience and Biotechnology, College of Life Science, Institute of Biotechnology, Sejong University, Kwangjingu, Kunjadong, Seoul 143-747, Korea; 2 Department of Life Science, University of Seoul, Dongdaemungu, Jeonnong-dong, Seoul 130-743, Korea

## Abstract

**Introduction:**

Estrogen receptor (ER) β is predicted to play an important role in prevention of breast cancer development and metastasis. We have shown previously that ERβ inhibits hypoxia inducible factor (HIF)-1α mediated transcription, but the mechanism by which ERβ works to exert this effect is not understood.

**Methods:**

Vascular endothelial growth factor (VEGF) was measured in conditioned medium by enzyme-linked immunosorbent assays. Reverse transcription polymerase chain reaction (RT-PCR), Western blotting, immunoprecipitation, luciferase assays and chromatin immunoprecipitation (ChIP) assays were used to ascertain the implication of ERβ on HIF-1 function.

**Results:**

In this study, we found that the inhibition of HIF-1 activity by ERβ expression was correlated with ERβ's ability to degrade aryl hydrocarbon receptor nuclear translocator (ARNT) via ubiquitination processes leading to the reduction of active HIF-1α/ARNT complexes. HIF-1 repression by ERβ was rescued by overexpression of ARNT as examined by hypoxia-responsive element (HRE)-driven luciferase assays. We show further that ERβ attenuated the hypoxic induction of VEGF mRNA by directly decreasing HIF-1α binding to the VEGF gene promoter.

**Conclusions:**

These results show that ERβ suppresses HIF-1α-mediated transcription via ARNT down-regulation, which may account for the tumour suppressive function of ERβ.

## Introduction

Estrogen plays a key role in the pathogenesis of breast cancer [1]. The cellular response to estrogen is mediated by two estrogen receptor (ER) isoforms, ERα and ERβ [2]. ER is the primary target for chemoprevention and endocrine therapy in breast cancer and provides prognostic and predictive information about tumour response to endocrine treatment [3]. A series of reports strongly indicated that estrogens, via ERα, stimulate proliferation and inhibit apoptosis [4,5], whereas ERβ opposes the proliferative effect of ERα *in vitro *[6,7]. The alteration of the intracellular ERα/ERβ ratio affects the estrogen-induced cellular response [8]. In addition to its role in modulating ERα-mediated regulation, ERβ also has distinct functions. Expression of ERβ significantly reduced cancer cell proliferation and tumour growth in severe combined immunodeficient mice [9]. ERβ inhibited proliferation of colon cancer cells [10]. It was suggested that the loss of ERβ expression may be one of the events leading to cancer development [11].

Hypoxia regulates a set of cellular functions, such as increased angiogenesis, energy metabolism, and erythropoiesis [12]. The adaptive response to hypoxia is controlled primarily by hypoxia-inducible factors (HIFs), which are master regulators of hypoxic gene expression and oxygen homeostasis [13-15]. HIF-1 plays a role in the physiologic regulation of a number of genes, such as vascular endothelial growth factor (VEGF), erythropoietin, and glucose transporter-1 expression in various tissues [16-18]. HIF-1 functions as a heterodimer, comprised of an oxygen-labile α-subunit and a stable β-subunit, also referred to as aryl hydrocarbon receptor nuclear translocator (ARNT) [15]. The HIF-1α subunit is degraded through a proteasome pathway under normoxia, whereas ARNT is constitutively expressed and located in the nucleus. At low oxygen levels, stabilized HIF-1α translocates to the nucleus, where the functionally active HIF-1α/ARNT complex activates the transcription of target genes after binding to cognate hypoxia-responsive elements (HRE) [19]. ARNT expression levels constitute important determinants of hypoxia responsiveness [20]. In addition to its role in the hypoxic pathway, ARNT interacts and functions as a potent coactivator of both ERα- and ERβ-dependent transcription [21]. The C-terminal domain of ARNT is essential for the transcriptional enhancement of ER activity [2]. ARNT is also required for aryl hydrocarbon receptor (AhR) function in 2,3,7,8-tetrachlorodibenzo-p-dioxin signalling [22]. Sequestering ARNT, by using a truncated AhR, blocks the hypoxia and ER signalling pathways [23]. The regulation of ARNT is implicated to have a significant impact on hypoxia and estrogen signalling pathways.

We recently reported that ERβ inhibits HIF-1α-mediated transcription [24]. However, the mechanism of ERβ on hypoxia-induced transcription is unknown. In this study, we show that ERβ significantly decreases the hypoxic induction of VEGF mRNA by inhibiting HIF-1-mediated transcription via ARNT downregulation providing mechanistic evidence for the anti-angiogenic effect of ERβ.

## Materials and methods

### Materials

17-β-estradiol (E2) and 2,3,7,8-tetrachlorodibenzo-p-dioxin (TCDD) (Supelco, Bellefonte, PA, USA) were purchased from Sigma (St. Louis, MO, USA) and dissolved in 100% ethanol. ICI182,780 (ICI) was obtained from ZENECA Pharmaceuticals (Tocris, UK). MG132 (Sigma) was dissolved in dimethyl sulfoxide. All of the compounds were added to the medium such that the total solvent concentration was never higher than 0.1%. An untreated group served as a control. Anti-ERβ was purchased from GeneTex (GTX110607, Irvine, CA, USA). Anti-ARNT and anti-HIF-1α were obtained from BD Biosciences (San Jose, CA, USA). Anti-β-actin and anti-ubiquitin were purchased from Sigma.

### Cell culture and hypoxic conditions

Hep3B and Human embryonic kidney 293 (HEK293) cells were maintained in phenol red-free Dulbecco's modified Eagle medium (DMEM) supplemented with 10% fetal bovine serum (FBS). MCF-7 and PC3 cells were maintained in phenol red-free RPMI 1640 medium supplemented with 10% FBS. Cells were grown at 37°C in a humidified atmosphere of 95% air/5% CO_2 _and fed every two to three days. Before treatment, the cells were washed with phosphate-buffered saline and cultured in DMEM/5% charcoal-dextran stripped FBS (CD-FBS) for two days to eliminate any estrogenic source before treatment. All treatments were done with DMEM/5% CD-FBS. We used 10 nM E2, unless otherwise noted. For the hypoxic condition, cells were incubated at a CO_2 _level of 5% with 1% O_2 _balanced with N_2 _using a hypoxic chamber (Forma, Costa Mesa, CA, USA).

### Plasmids

The hERβ expression vector was kindly provided by Dr. Mesut Muyan (University of Rochester Medical School, USA). The HRE-Luc reporter plasmid contains four copies of the erythropoietin HRE, the SV40 promoter, and the luciferase gene. Green fluorescent protein (GFP) tagged HIF-1α (GFP-HIF-1α) vector was kindly provided by Dr. Kyu-Won Kim (Seoul National University, Korea). The plasmid His-tagged ubiquitin (His-Ub) was constructed by inserting a single copy of the Ub gene (76 amino acids) into pcDNA3.1/HisC (Invitrogen, Carlsbad, CA, USA).

### Transient transfection and luciferase assay

HEK293 and MCF-7 were transiently transfected with plasmids by using the polyethylenimine (PEI; Polysciences, Warrington, PA, USA). Luciferase activity was determined 24 or 48 h after treatment with an AutoLumat LB953 luminometer using the luciferase assay system (Promega Corp., Madison, WI, USA) and expressed as relative light units. The means and standard deviations (SD) of three replicates are shown for the representative experiments. All transfection experiments were repeated three or more times with similar results. PC3 cells were transfected transiently with Lipofectamine 2000 (Invitrogen) and On-Target Plus SMARTpool siRNAs (Dharmacon, Lafayette, CO, USA) for ERβ Nontargeting pools were used as negative controls.

### Reverse transcription (RT)-Polymerase chain reation (PCR)

Total RNA was extracted using Trizol reagent (Invitrogen) according to the manufacturer's instruction. RNA pellets were dissolved in diethylpyrocarbonate-treated water. To synthesize first strand cDNA, 3 μg total RNA was incubated at 70°C for five minutes with 0.5 μg of random hexamer and deionized water (up to 11 μl). The reverse transcription reaction was performed using 40 units of M-ML reverse transcriptase (Promega) in 5× reaction buffer (250 mmol/l Tris-HCl; pH 8.3, 375 mM KCl, 15 mM MgCl_2_, 50 mM DTT), RNase inhibitor at 1 unit/μl, and 1 mM dNTP mixtures at 37°C for 60 minutes. The resulting cDNA was added to the PCR reaction mixture containing 10× PCR buffer (100 mM Tris-HCl, pH 8.3, 500 mM KCl, 15 mM MgCl_2_), 25 units of rTaq polymerase (TakaRa, Shiga, Japan), 4 μl of 2.5 mM dNTP mixtures, and 10 pmol of primers each. The resulting cDNA samples were amplified using Mastercycler gradient (Eppendorf, Hauppauge, NY, USA). The primers used were: VEGF sense primer, 5'- ATGAACTTTCTGCTCTCTGG -3'; anti-sense primer, 5'-TCATCTCTCCTATGTGCTGGC-3'; β-actin sense primer, 5'-CCTGACCCTGAAGTACCCCA-3'; anti-sense primer, 5'-CGTCATGCAGCTCATAGCTC-3'. Quantitative real-time PCR (qPCR) was used to detect cytochrome p450 (CYP) 1A1. qPCR was performed using iQ™ SYBR Green Supermix (Bio-Rad, Hercules, CA, USA). The primers used were: β-actin sense primer, 5'- CAAATGCTTCTAGGCGGACTATG-3'; anti-sense primer, 5'- TGCGCAAGTTAGGTTTTGTCA -3'; CYP 1A1 sense primer, 5'- TAGACACTGATCTGGCTGCA-3'; anti-sense primer, 5'- GGGAAGGCTCCATCAGCATC-3'; ERβ sense primer, 5'- GTCAGGCATGCGAGTAACAA-3'; anti-sense primer, 5'-GGGAGCCCTCTTTGCTTTTA-3'. A final volume was 25 μl, and an iCycler iQ Real-time PCR Detection System (Bio-Rad) was used for qPCR. The amplification data were analyzed by iQ™5 optical system software version 2.1 and calculated using the ΔΔC_T _method. The ΔΔC_T _method was used to calculate relative mRNA expression. The relative target gene expression was calculated using 2^-ΔΔCT^, where ΔC_T _= target C_T _- control C_T_, ΔΔC_T _=ΔC_T _target - ΔC_T _calibrator.

### VEGF ELISA

After hypoxic exposure, culture medium was removed and stored at -80°C until assayed. VEGF concentrations were determined using an ELISA kit (R&D Systems, Minneapolis, MN, USA) according to the manufacturer's instructions. Samples from two different experiments were analyzed in triplicate.

### Western blot analysis

Protein extracts were isolated in lysis buffer (150 mM NaCl, 50 mM Tris-HCl (pH 8.0), 5 mM EDTA, 1% Nonidet P-40, 0.5% deoxycholate, 1% SDS) with protease inhibitor cocktail (Sigma) on ice for 1 h and then centrifuged for 20 minutes at 13,000 × g. Supernatant was collected and protein concentrations were measured using the Bradford method (Bio-Rad). Proteins were dissolved in sample buffer and boiled for five minutes prior to loading onto a polyacrylamide gel. After SDS-PAGE, proteins were transferred to a polyvinylidene difluoride membrane, blocked with 5% nonfat dry milk in Tris-buffered saline containing 0.1% Tween-20 (TBST) for 1 h at room temperature. The membranes were incubated for 2 h at room temperature with antibody. Equal lane loading was assessed using β-actin monoclonal antibody (Sigma). After washing with TBST, blots were incubated with 1:5,000 dilution of the horseradish peroxidase conjugated-secondary antibody (Zymed, San Francisco, CA, USA), and washed again three times with TBST. The transferred proteins were visualized with an enhanced chemiluminescence detection kit (Amersham Pharmacia Biotech, Buckinghamshire, UK).

### Immunoprecipitation

Two hundred micrograms of the cell lysates were mixed with 1 μg of antibody and incubated overnight at 4°C with constant rotation. To recover immunoprecipitated complexes, 150 μl of protein A-sepharose, diluted 1:1 in PBS, were then added to the samples and incubated on ice for an additional two to four hours with constant rotation. The beads were pelleted by centrifugation and the bound proteins were eluted by incubation in 5X SDS loading buffer for five minutes by boiling. The eluted proteins were analyzed by immunoblot analysis.

### Chromatin immunoprecipitation (ChIP) assay

Hep3B cells exposed to hypoxia as indicated in the figure legends were cross-linked by adding formaldehyde to a final concentration of 1% and incubating at 37°C for 10 minutes. Cells were washed twice with ice-cold phosphate-buffered saline. Cells were washed sequentially in Buffer 1 (0.25% Triton X-100, 10 mM EDTA, 0.5 mM EGTA, 10 mM HEPES, pH 6.5) and Buffer 2 (200 mM NaCl, 1 mM EDTA, 0.5 mM EGTA, 10 mM HEPES, pH 6.5). Cells were pelleted at 4°C and resuspended in 0.3 ml of cell lysis buffer (50 mM Tris-HCl pH 8.1, 10 mM EDTA, 1% SDS) containing complete protease inhibitor mixture (Sigma). Cell lysates were sonicated to yield DNA fragments ranging in size from 200 to 900 bp. Samples were centrifuged for 15 minutes at 4°C. Supernatants were diluted 10-fold to a final solution containing 20 mM Tris-HCl (pH 8.1), 1% Triton X-100, 2 mM EDTA, 150 mM NaCl, and complete protease inhibitor mixture. Eluates were then incubated with 2 μg of HIF-1α antibody (BD Biosciences) overnight at 4°C followed by the addition of 50 μl of 50% slurry of protein A or protein G-Sepharose and incubated at 4°C for an additional two hours. Sepharose beads were pelleted and washed sequentially for 10 minutes each in TSE I (0.1% SDS, 1% Triton X-100, 2 mM EDTA, 20 mM Tris, pH 8.1, 150 mM NaCl), TSE II (0.1% SDS, 1% Triton X-100, 2 mM EDTA, 20 mM Tris, pH 8.1, 500 mM NaCl), and Buffer III (0.25 M LiCl, 1% Nonidet P-40, 1% deoxycholate, 1 mM EDTA, 10 mM Tris, pH 8.1). Beads were washed another three times in Tris-EDTA, pH 8.0, and protein-DNA complexes were eluted in 300 μl of elution buffer (1% SDS, 0.1 M NaHCO3). Chemical cross-links were reversed by heating the samples overnight at 65°C; the DNA was separated from protein and used as a template for PCR reactions. The yield of target region DNA in each sample after ChIP was analyzed by conventional PCR. The following primers were used for PCR analysis: VEGF promoter -1215 to -881 (HRE-containing region), forward 5'- TTGGGCTGATAGAAGCCTTG -3' and reverse 5'- TGGCACCAAGTTTGTGGAGC -3'. For qPCR, standard curves were generated by serially diluting an input chromatin sample. DNA regions were amplified using the following primers: VEGF promoter -1077 to -975(HRE-containing region), forward 5'-CCTCAGTTCCCTGGCAACATCTG-3' and reverse 5'-GAAGAATTTGGCACCAAGTTTGT-3' [GenBank:AF095785.1].

### Statistical analysis

Values shown represent the mean ± SD. Statistical analysis was performed by Student's *t*-test, and a *P-*value <0.05 was considered significant.

## Results

### ERβ decreases HIF-1α mediated gene transcription

**Figure 1:**
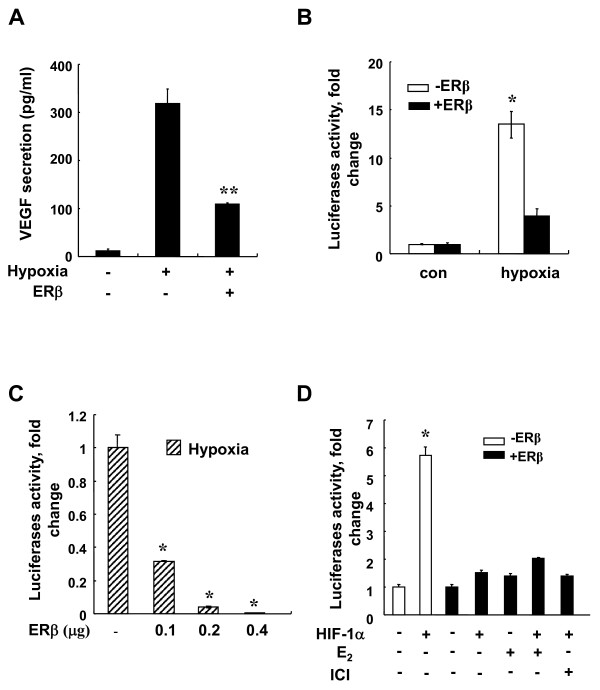
**ERβ decreases HIF-1α mediated gene transcription**. **(**a**)** HEK293 cells in 24-well plates were transfected with hERβ (0.2 μg). At 24 h post-transfection, cells were incubated for 48 h under hypoxic conditions. Culture medium from untransfected HEK293 cells and cells transfected with hERβ plasmids were analyzed using VEGF ELISA kit. Values represent the mean ± S.D. (N = 3). ** *P *<0.01. **(**b**)** At 24 h post-transfection of hERβ and HRE-Luc (0.5 μg), cells were incubated for 24 h under normoxic or hypoxic conditions. After incubation, luciferase expression was determined. Values represent the mean ± S.D. (N = 3). * *P *<0.05. **(**c**)** Cells were transfected with HRE-Luc reporter with or without the expression vector for 0.1 to 0.4 μg hERβ. At 24 h post-transfection, cells were incubated for 24 h under normoxic or hypoxic conditions. After incubation, luciferase expression was determined. Values represent the mean ± S.D. (N = 3). * *P *<0.05. **(**d**)** Cells were transfected with HRE-Luc reporter and hHIF-1α (0.2 μg) with or without the expression vector for hERβ (0.2 μg). At 24 h post-transfection, cells were left untreated or treated with 10 nM E2 or 1 μM ICI and incubated for 24 h under normoxic conditions. After incubation, luciferase expression was determined. Values represent the mean ± S.D. (N = 3). * *P *<0.05. All experiments were repeated at least three times.

We have previously reported that overexpression of ERβ suppresses hypoxia-induced endogenous VEGF mRNA [24]. To determine whether ERβ affects hypoxia-induced VEGF secretion, HEK293 cells were transfected with vector control or ERβ and exposed to hypoxia for 48 h. VEGF secretion was measured by ELISA. As shown in Figure 1A, expression of ERβ significantly decreased VEGF secretion under hypoxic condition. To further characterize the molecular details of ERβ inhibition of hypoxia-induced transcription activation, we studied the effect of ERβ expression on HIF-1α-mediated gene transcription by using an HRE-driven reporter gene. HEK293 cells were transfected with an HRE-Luc plasmid with or without an expression vector for ERβ under hypoxia. As shown in Figure 1B, C, the HRE-driven luciferase reporter was markedly activated by hypoxia, whereas ERβ significantly inhibited this hypoxic activation in a dose dependent manner. Next, we examined whether the inhibition was dependent on HIF-1α by using GFP-HIF-1α, which showed increased stability enough to carry out HIF-1 functional analyses under normoxia. The expression of ERβ significantly decreased the transcriptional activity of HIF-1α under normoxia (Figure 1D). However, the E2 or ER antagonist, ICI, did not additionally affect this suppression (Figure 1D). This shows that unoccupied ERβ itself serves as a negative regulator of HIF-1.

### HIF-1 suppression by ERβ is due to ARNT degradation

**Figure 2:**
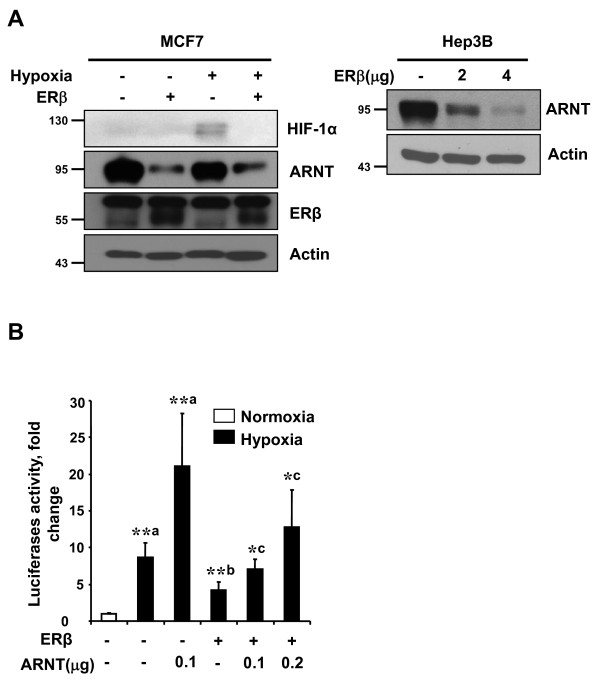
**HIF-1 suppression by ERβ is due to ARNT degradation**. **(**a**)** Hep3B or MCF-7 cells in six-well plates were transfected with hERβ. At 36 h post-transfection, total protein extracts were immunoblotted as indicated. **(**b**)** HEK293 cells in 24-well plates were cotransfected with HRE-Luc reporter (0.5 μg) and hERβ (0.2 μg) or ARNT plasmid (0.1 or 0.2 μg) as indicated. At 24 h post-transfection, cells were incubated under hypoxic conditions for 24 h. After incubation, luciferase expression was determined. Values represent the mean ± S.D. (N = 3). **a *P *<0.005 vs. con; **b *P *<0.005 vs. Hypoxia; *c *P *<0.05 vs. Hypoxia+ERβ. All experiments were repeated at least three times.

**Figure 3:**
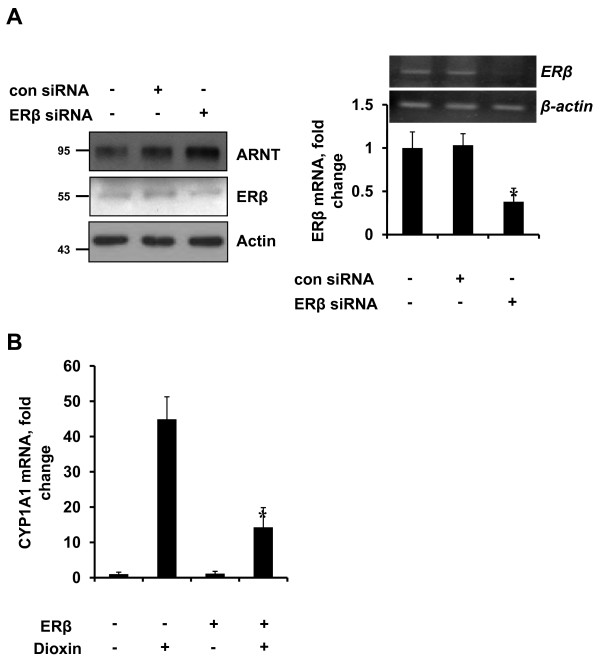
**Effects of ERβ on ARNT and CYP1A1 expression level**. **(**a**)** PC3 cells in six-well plates were transfected with control siRNA or ERβ SMARTpool siRNA. At 48 h post-transfection, total protein extracts were immunoblotted as indicated (left panel). Knockdown of ERβ mRNA was also verified by RT-qPCR. Both quantified and gel electrophoretic results of qPCR products are shown (right panel). * *P *<0.001. **(**b**)** MCF7 cells in six-well plates were transfected with hERβ (2 μg). At 24 h post-transfection, cells were treated for 24 h with 1 nM dioxin as indicated. Total RNA were extracted and expression of CYP1A1 was analyzed by qPCR. * *P *<0.0005.

Association of HIF-1α with ARNT, forming a heterodimeric complex, is required for it to bind to the HRE of target genes and its subsequent transactivation function [18]. As adequate levels of ARNT protein are required for the formation of the active HIF-1 heterodimeric complex, we determined the effect of ERβ on the expression of ARNT. To our surprise, we observed that ERβ down-regulates the ARNT protein levels in Hep3B and MCF-7 cells transfected with ERβ (Figure 2A). In addition, ARNT overexpression effectively rescued HIF-1 repression by ERβ (Figure 2B). These results imply that ERβ induced HIF-1 transrepression is attributed to the down-regulation of ARNT. The involvement of ERβ modulation of ARNT protein level was also confirmed after knockdown of ERβ using RNA interference. As shown, ARNT protein levels were increased when the expression of ERβ was repressed in PC3 cells. Knockdown of ERβ mRNA by ERβ-siRNA were validated by qPCR (Figure 3A). ERβ expression in cell lines used in this study is shown in Supplementary Figure S1 in Additional file 1.

To further confirm the decrease in ARNT expression by ERβ, we have examined suppression of AhR activity which exerts its effect by formation of heterodimer with ARNT. Dioxin-occupied AhR/ANRT complex is well known to induce CYP1A1 [25]. As shown, ERβ expression significantly suppressed dioxin induced CYP1A1 expression in MCF-7 cells (Figure 3B). The same effects were observed in rat hepatocytes (Supplementary Figure S2 in Additional file 2).

### Effects of ERβ on ARNT binding with HIF-1α

**Figure 4:**
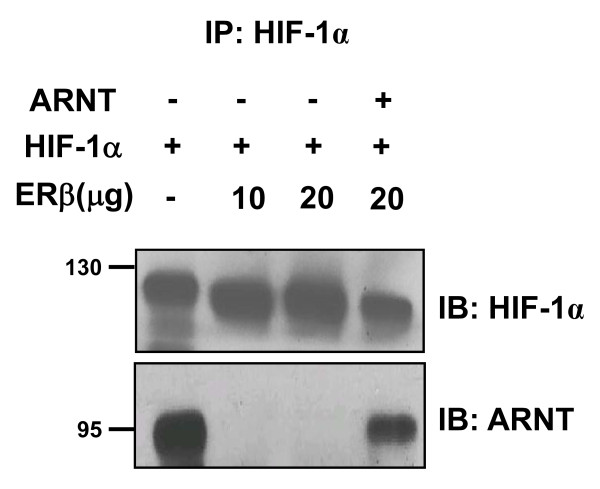
**Effects of ERβ on ARNT binding with HIF-1α**. HEK293 cells in 100 mm^2 ^dish were cotransfected with GFP-HIF-1α (10 μg) and hERβ (10 or 20 μg) or ARNT plasmid (10 μg) as indicated. Cell lysates were immunoprecipitated with anti-HIF-1α antibody and precipitated proteins were analyzed by immunoblots with anti-HIF-1α (top) or anti-ARNT antibody (bottom). Shown are representative results from two to three separate experiments.

Our data strongly suggest that ERβ decreases HIF-1α mediated gene transcription through ARNT down-regulation. To further examine the functional consequences resulting from the degradation of ARNT protein, the formation of HIF-1α/ARNT complexes was assessed in HEK293 cells. As shown in Figure 4, GFP-HIF-1α/ARNT complex levels were significantly decreased by the overexpression of ERβ under normoxia, as determined by coimmunoprecipitation. In addition, ARNT overexpression effectively recovered HIF-1α binding to ARNT (Figure 4), showing that ARNT degradation by ERβ is followed by the reduction of HIF-1α/ARNT complex formation. In Figure 4, we detected no ARNT protein upon ERβ expression in contrast to the low levels of ARNT protein in Figure 2A. The difference in levels of ARNT protein between Figures 2A and 4 is probably due to the efficiency of technique used in detection.

### ERβ degrades ARNT via the ubiquitin proteasome system

**Figure 5:**
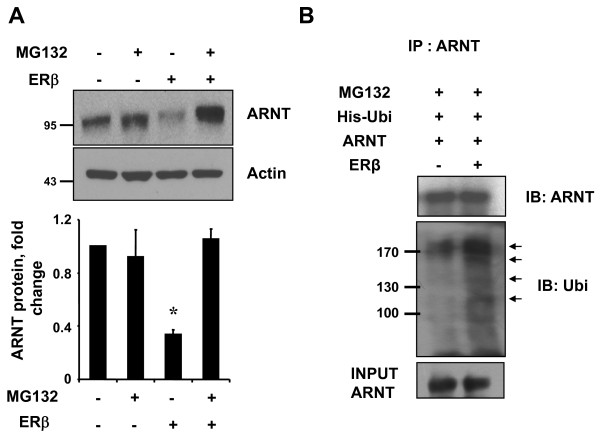
**ERβ degrades ARNT via the ubiquitin-proteasome system**. **(**a**)** HEK293 cells were transfected with hERβ. At 36 h post-transfection, cells were treated with 10 μM MG132 for 12 h. The total protein extracts were immunoblotted with ARNT antibody and reprobed with actin antibody. Values represent the mean ± S.D. (N = 3). * *P *<0.01. All experiments were repeated at least three times. **(**b**)** HEK293 cells in 100Q dish were transfected with His-Ubi (10 μg) and ARNT plasmid (10 μg) or hERβ (10 μg) as indicated. At 36 h post-transfection, cells were treated with 10 μM MG132 for 12 h. Immunoprecipitated ARNT or His-Ubi-conjugated ARNT was detected using anti-ARNT or anti-ubiquitin antibody, respectively. Arrows indicate the ubiquitinated ARNT protein bands. Molecular weight markers are as shown. The molecular mass of the human ARNT is 95 kDa. Shown are representative results from two separate experiments.

The ubiquitin-proteasome pathway is responsible for many regulatory proteins. To examine the involvement of the proteasomal pathway in ERβ-induced degradation of ARNT, HEK293 cells were transfected with ERβ and treated with or without 10 μM of the proteasome inhibitor, MG132 for 12 h. We analyzed the lysates using Western blots. As shown in Figure 5A, MG132 significantly blocked ARNT degradation by ERβ, suggesting that ERβ degrades ARNT via the proteasomal pathway. Protein ubiquitination is a signal for targeted recognition and proteolysis by proteasome [26]. To assess ubiquitination of ARNT by ERβ, cell lysates from HEK293 cells transfected with ERβ, ARNT, and His-Ubi were immunoprecipitated with anti-ARNT antibody and then analyzed by Western blot using anti-ubiquitin antibodies. As shown in Figure 5B, ubiqutination of the ARNT protein was enhanced by ERβ expression, indicating that this process is mediated through the ubiquitin-proteasome pathway.

### ERβ decreases the hypoxic induction of VEGF by reducing the recruitment of HIF-1 to the hypoxia-dependent VEGF promoter

**Figure 6:**
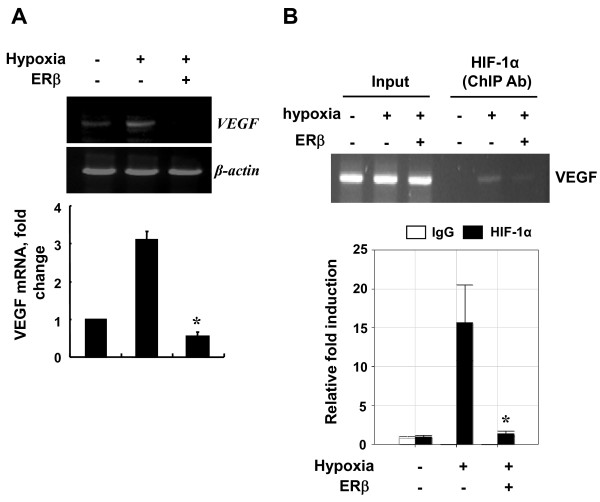
**ERβ decreases the hypoxic induction of VEGF by reducing the recruitment of HIF-1 to the hypoxia-dependent VEGF promoter**. **(**a**)** Hep3B cells in six-well plates were transfected with hERβ (2 μg). At 24 h post-transfection, cells were incubated for 24 h under normoxic or hypoxic conditions. Total RNA from untransfected Hep3B cells and cells transfected with hERβ plasmids were analyzed using VEGF-specific primers, as described in the Materials and Methods. Values represent the mean ± S.D. (N = 3). * *P *<0.05. All experiments were repeated at least three times. **(**b**)** Hep3B cells were transfected with hERβ using the PEI method. At 24 h post-transfection, cells were incubated for 24 h under hypoxic conditions. After incubation, cells were subjected to ChIP analysis with HIF-1α or control IgG antibody. Shown here are immunoenriched DNA samples amplified using conventional PCR (top) and quantified results by qPCR (bottom) for an HIF-1α binding site on the VEGF promoter from three separate experiments.

We have previously reported that ERβ decreases VEGF mRNA in HEK293 cells [24]. To examine the possibility that ERβ modulates the expression of VEGF in other cells, Hep3B cells were transfected with the expression vector for ERβ and exposed to hypoxia. The hypoxic induction of VEGF mRNA was significantly blocked by the overexpression of ERβ in Hep3B cells (Figure 6A).

HIF functions by binding to the HREs present in the promoter of hypoxic genes [27]. To investigate whether ERβ results in reduced HIF-1 recruitment to the VEGF promoter, we performed ChIP assays on the VEGF promoter in Hep3B cells. As shown in Figure 5B, association of HIF-1α at the VEGF promoter after ERβ overexpression was significantly decreased compared with that in hypoxia-treated cells (Figure 6B). This shows that ERβ induced the down-regulation of the HIF-1 target gene expression resulting from a reduction in the level of HIF-1 binding to the VEGF promoter.

## Discussion

In this study, we sought to determine whether ERβ regulates HIF-1α-mediated transcription by targeting ARNT. Using a reporter-based assay, we found that ERβ decreased HIF-1α-mediated transcription. Hypoxic induction of endogenous VEGF was blocked by ERβ expression. This repression is due to ERβ-induced down-regulation of ARNT via ubiquitination processes. Overexpression of ARNT rescued HIF-1 repression by ERβ. Two important aspects of our study are that it provides a mechanistic explanation for ERβ as a tumour suppressor and a distinct function for unliganded ERβ in post-translational regulation. The tumour-suppressive role of ERβ in cancer biology currently is being widely studied [8]. ERβ inhibits angiogenesis and growth of T47D breast cancer xenografts [9]. Coradini *et al*. reported that VEGF synthesis under hypoxia was reduced in ERβ-expressing MDA-MB231 breast cancer cells in contrast to MCF-7 cells containing both the ERα and ERβ isoforms [28]. A very recent study by Maik *et al*. showed that ligand-bound ERβ impedes prostate cancer epithelial-mesenchymal transition by destabilizing HIF-1α and impeding HIF-1 mediated transcription of VEGF [29]. Our data showed that ERβ suppresses HIF-1 activity and inhibits angiogenesis related gene expression by targeting ARNT. The detailed complex regulatory mechanisms of ERβ targeting HIF-1 components to proteasome need to be delineated.

ARNT plays a critical role in the transcriptional response to hypoxia and inactivation of ARNT is sufficient to suppress HIF target gene induction [30,31]. Reducing the cellular levels of ARNT significantly attenuated the transcriptional response of ERβ [2]. These results, along with our data, indicate that ERβ-ARNT crosstalk is an important regulatory constituent responsible for the inhibitory effects of ERβ in hypoxia response, although the gap between ERβ and proteasomal degradation of ARNT still needs to be investigated. Pongratz group has reported the role of ARNT as a modulator of ERs. C-terminal part of ARNT interacts with the ER ligand binding domain [2]. Since ubiquitination by proteins such as carboxyl terminus of Hsc 70 interacting protein, a regulatory subunit of 26 S proteasome SUG1/TRIP1 and E6-AP ubiquitin ligase promotes ligand-induced degradation of ERβ [32], ERβ-ARNT co-regulator complexes may contain proteins inducing ARNT degradation. Despite the extensive study on HIF-1α regulation, little is known about ARNT regulation. ARNT is present at constituent levels with a short half life of 4.84 h. There are other tumour inhibitory substances targeting ARNT degradation such as curcumin, a major component of turmeric [33]. Curcumin induces degradation of ARNT via oxidation and ubiquitination. Further work will reveal the identity of protein complexes responsible for ARNT degradation.

The modulation of hypoxic transcription is not confined to the ERβ. Nuclear hormone receptors affecting hypoxic activity are reported by several groups [34-39]. E2 protects against hypoxic/ischemic white matter damage in the neonatal rat brain [40] and hypoxia-induced hepatocyte injury through ER-mediated up-regulation of Bcl-2 [41]. Hypoxia either enhances or inhibits transcriptional activity of glucocorticoid receptors [42], androgen receptors [43], ERs [24,44-47], and peroxisome proliferator-activated receptors [48-51] depending on the experimental systems. Increased glucocorticoid sensitivity after hypoxia exposure has been observed [52], suggesting that hypoxia may influence the inflammation process as well. Despite the importance of understanding the crosstalk between nuclear receptors and hypoxia-responsive pathways, which will greatly aid the progress of cancer biology, the mechanism of the crosstalk is not yet understood. It is possible that common co-regulator(s) may be involved rather than specific co-regulators for each nuclear hormone receptor in hypoxia and nuclear receptor crosstalk. HIF-1 transactivates and down-regulates ERα [45,46], so the co-regulator(s) may contain proteasome activity. Recent reports showed that the carboxy terminus of 70-kDa heat shock protein-interacting protein, which can degrade ERα, contains a dual function as an ubiqutin ligase and tumour suppressor [53].

Another interesting aspect of our result is that the effect of ERβ on hypoxia-mediated response is independent of ligand. Unoccupied ERα is known to be associated with DNA, even before ligand exposure. ChIP data showed that unliganded ERα is assembled with transcription activation complexes for tumour necrosis factor-α induction [54]. Maynadier *et al*. reported that unliganded ERα inhibits cell growth through interaction with the cyclin-dependent kinase inhibitor p21WAF1 [55]. Lazennec *et al*. showed that overexpression of ERβ inhibited E2 induced cell proliferation even at low E2 concentration [56] indicating that the effect of is not dependent on ligand. We and others have reported increased recruitment of SRC-1 and CBP to ERβ by liganded independent manner by EGF, oncogene ras and hypoxia [24,57,58]. We envision that unliganded ERβ recruits protein complex containing proteasomal degradation function although we cannot completely preclude the possibility that in vitro overexpression system have aberrantly activated ERβ.

## Conclusions

In conclusion, our study demonstrated that ERβ degrades ARNT via the ubiquitin-proteasome system leading to HIF-1 suppression. The ERβ/HIF-1α/ARNT pathway may play an important role in cancer progression. These findings suggest that HIF-1 suppression by ERβ may represent a potential therapeutic target in treating patients with ER-associated cancer.

## Abbreviations

ARNT: aryl hydrocarbon receptor nuclear translocator; ER: estrogen receptor; HIF: hypoxia-inducible factor; HRE: hypoxia-responsive element; RT-PCR: reverse transcription polymerase chain reaction; TCDD: 2,3,7,8-tetrachlorodibenzo-p-dioxin; VEGF: vascular endothelial growth factor.

## Competing interests

The authors declare that they have no competing interests.

## Authors' contributions

WL, YP, JC, JP, CP, YP and HP carried out experiments and drafted the manuscript. YJL conceived of the study, participated in its design, coordination and interpretation of the results, and finalized the manuscript. All authors read and approved the final manuscript.

## Supplementary Material

Additional file 1 **Supplemental Figure S1**. Expression level of ERβ in the following cells. MCF7, Hep3B and PC3 cells total protein extracts were immunoblotted with ARNT, ERβ and β-actin antibody. Click here for file

Additional file 2 **Supplemental Figure S2**. Effects of ERβ expression on CYP1A1 level. Hepa1c1c7 cells in six-well plates were transfected with hERβ (2 μg) or same amounts of empty vector. At 24 h post-transfection, cells were treated for 48 h with 1 nM dioxin as indicated. Total protein extractes were immunoblotted with ARNT and ERβ (left panel). Total RNA were extracted and expression of CYP1A1 was analyzed by qRT-PCR (right panel). The expression level of 18S rRNA was used for normalization. The primers used were: mouse Cyp1A1, 5'-TTCCTGTCCTCCGTTACCTG-3' and 5'-CCTGTCCTGACAATGCTCAA-3'; 18S rRNA, 5'-ACCGCAGCTAGGAATAATGGAATA-3'; 5'-CTTTCGCTCTGGTCCGTCTT-3. Click here for file
