# Supplementary figures and images for: Estrogen receptor beta inhibits transcriptional activity of hypoxia inducible factor-1 through the downregulation of arylhydrocarbon receptor nuclear translocator
Source: Breast Cancer Res. 2011 Mar 24;13(2):R32. doi: 10.1186/bcr2854 (PMC3219195; doi:10.1186/bcr2854)

## Slide 1
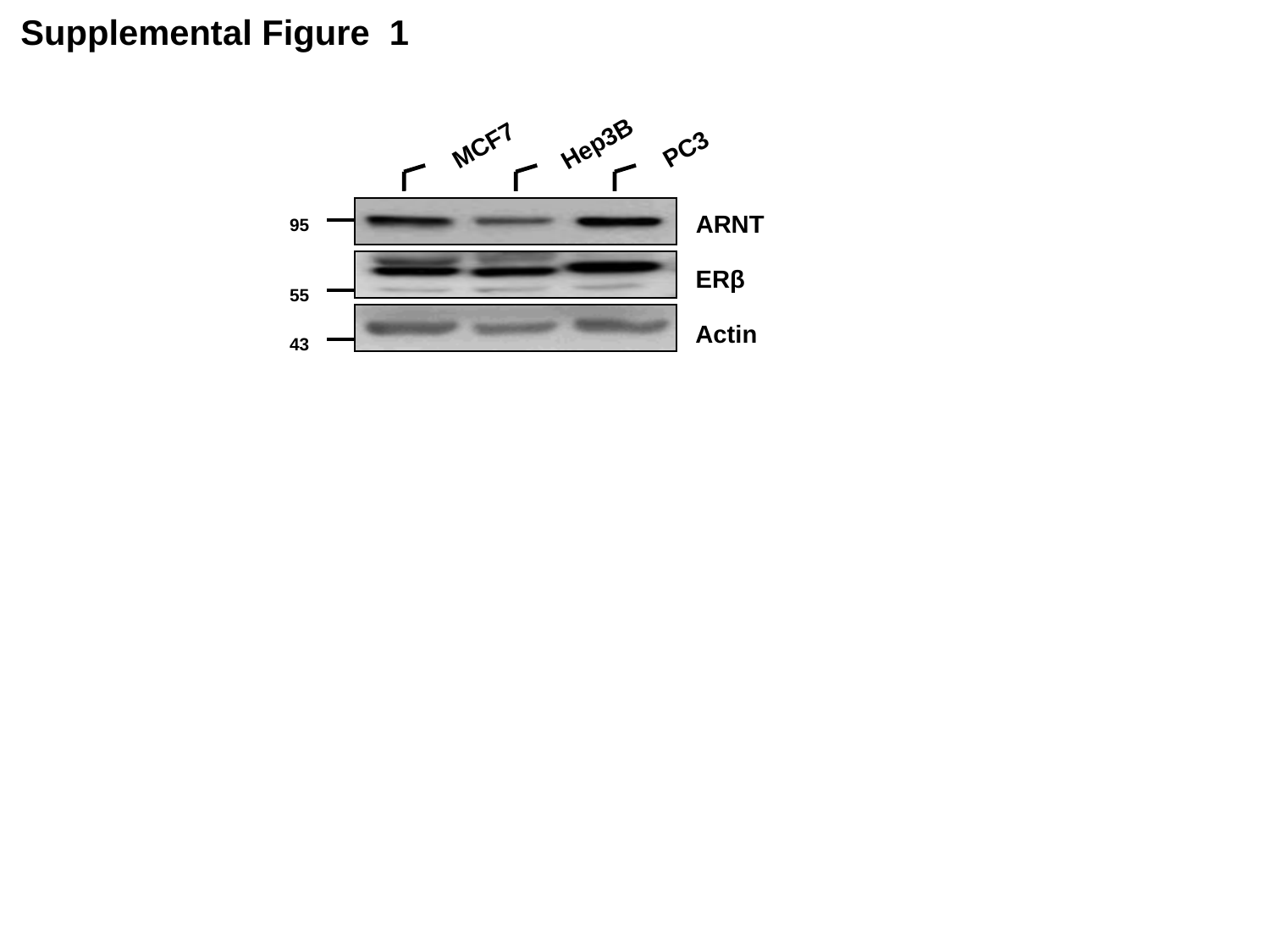

Supplemental Figure 1
Hep3B
MCF7
PC3
ARNT
95
ERβ
55
Actin
43

Supplement: Additional file 1 — Supplemental Figure S1. Expression level of ERβ in the following cells. MCF7, Hep3B and PC3 cells total protein extracts were immunoblotted with ARNT, ERβ and β-actin antibody. [file bcr2854-S1.PPTX]
