# Supplementary material for: Estrogen receptor beta inhibits transcriptional activity of hypoxia inducible factor-1 through the downregulation of arylhydrocarbon receptor nuclear translocator
Source: Breast Cancer Res. 2011 Mar 24;13(2):R32. doi: 10.1186/bcr2854 (PMC3219195; doi:10.1186/bcr2854)
Supplement: Additional file 2 — Supplemental Figure S2. Effects of ERβ expression on CYP1A1 level. Hepa1c1c7 cells in six-well plates were transfected with hERβ (2 μg) or same amounts of empty vector. At 24 h post-transfection, cells were treated for 48 h with 1 nM dioxin as indicated. Total protein extractes were immunoblotted with ARNT and ERβ (left panel). Total RNA were extracted and expression of CYP1A1 was analyzed by qRT-PCR (right panel). The expression level of 18S rRNA was used for normalization. The primers used were: mouse Cyp1A1, 5'-TTCCTGTCCTCCGTTACCTG-3' and 5'-CCTGTCCTGACAATGCTCAA-3'; 18S rRNA, 5'-ACCGCAGCTAGGAATAATGGAATA-3'; 5'-CTTTCGCTCTGGTCCGTCTT-3. [file bcr2854-S2.PPTX]

## Slide 1
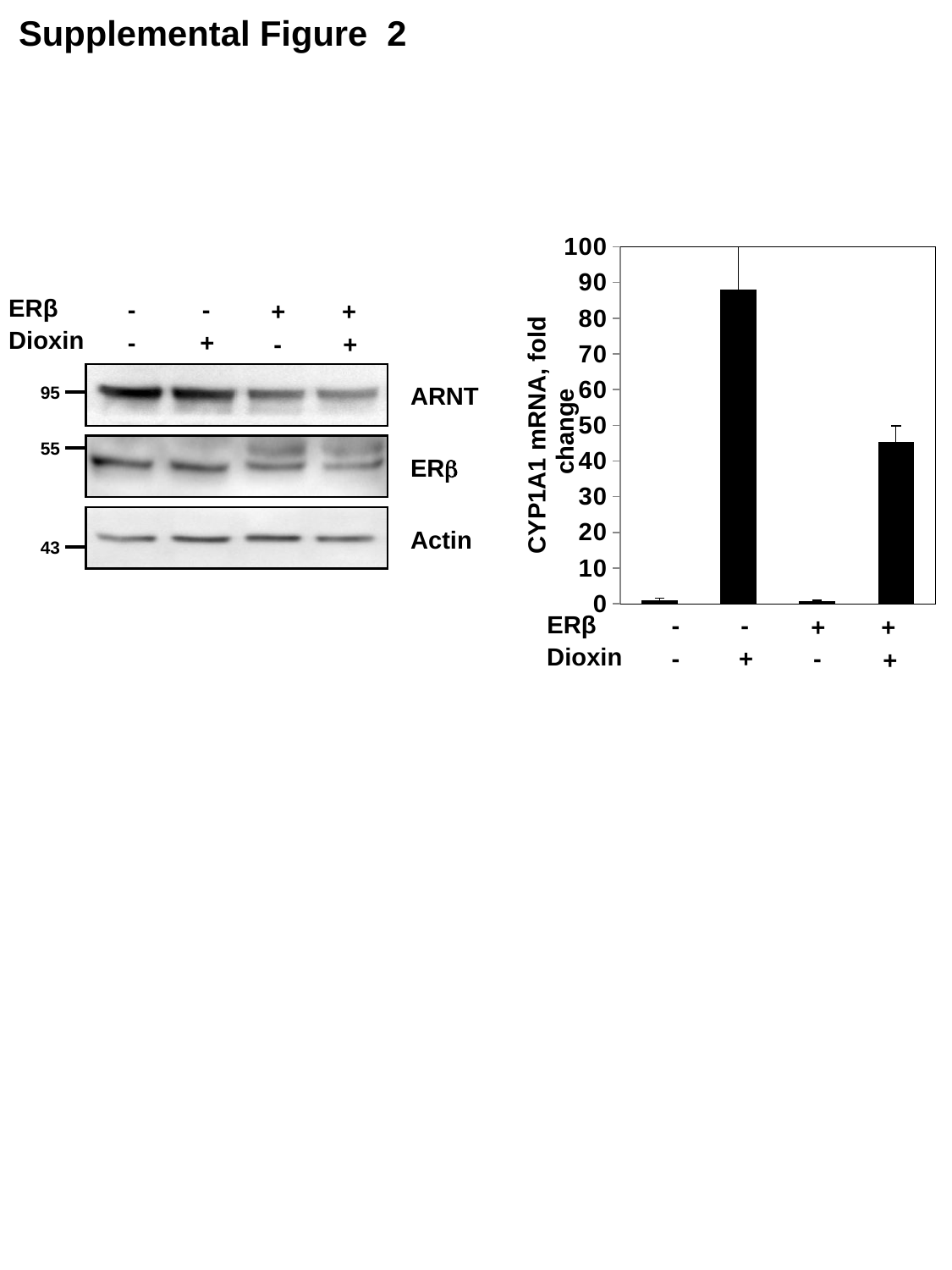

Supplemental Figure 2
### Chart
| Category | Cyp1a1 |
|---|---|
| EV - | 1.0 |
| EV + | 88.14 |
| ERb - | 0.6300000000000034 |
| ERb + | 45.42 |CYP1A1 mRNA, fold
change
ERβ
-
-
+
+
Dioxin
-
-
+
+
ERβ
-
-
+
+
Dioxin
-
+
-
+
ARNT
95
55
ERβ
Actin
43
